# Efficacy and Safety of Melatonin in Migraine Prophylaxis: A Systematic Review and Meta-Analysis of Randomized Controlled Trials

**DOI:** 10.1007/s11916-025-01461-5

**Published:** 2026-02-02

**Authors:** Moaz Elsayed Abouelmagd, Mohamed A. Aldemerdash, Abdallah Ahmad Khatatbeh, Ahmed S.A Osman, Abdallah Abbas, Salma Allam, Esraa M. AlEdani, Ahmed Aldemerdash, Teshamae S. Monteith

**Affiliations:** 1 Kasr-Alainy Faculty of Medicine, Cairo University, Cairo, Egypt; 2 Faculty of Medicine, Sohag University, Sohag, Egypt; 3 Faculty of Medicine, Ain Shams University, Cairo, Egypt; 4 Prince Al-Hussein bin Abdullah Hospital, Amman, Jordan; 5 Faculty of Medicine, Minia University, Minya, Egypt; 6 Medical Research Group of Egypt, Negida Academy, Arlington, MA USA; 7 Faculty of Medicine, Al-Azhar University, Damietta, Egypt; 8 Faculty of Medicine, Galala University, Suez, Egypt; 9 Faculty of Medicine, University of Basra, Basrah, Iraq; 10 Department of Neurology, Headache Division, Miami, FL USA

**Keywords:** Systematic review, Melatonin, Amitriptyline, Placebo, Migraine, Meta-analysis

## Abstract

**Background:**

Migraine is a chronic, disabling brain disorder. Melatonin, a circadian regulator with anti-inflammatory and antinociceptive actions, has been proposed for migraine prevention. We evaluated the efficacy and safety of melatonin for prophylaxis.

**Methods:**

We systematically searched PubMed, Cochrane, Scopus, Embase, and Web of Science (September 29, 2024) for randomised controlled trials (RCTs) comparing melatonin with placebo or other active drugs. Outcomes were analysed as change from baseline to last follow-up using mean differences (MD) or risk ratios (RR) with 95% confidence intervals (CI).

**Results:**

Nine RCTs (*n* = 788) were included. Versus placebo, melatonin reduced attack duration (MD -4.98 h; 95% CI -9.30 to -0.67; *p* = 0.02), headache days (MD -1.54 days; 95% CI -2.50 to -0.58; *p* < 0.01), headache severity (MD -2.08; 95% CI -2.91 to -1.26; *p* < 0.01), and analgesic use (MD -1.38; 95% CI -2.41 to -0.36; *p* < 0.01). Melatonin also increased the response rate (≥ 50% reduction in monthly headache frequency) (RR 1.38; 95% CI 1.11–1.70; *p* < 0.01) and improved sleep quality (PSQI: MD -1.64; 95% CI -2.85 to -0.42; *p* = 0.008) and disability (MIDAS: SMD − 4.07; 95% CI -5.45 to -2.69; *p* < 0.001). Compared with amitriptyline, melatonin was generally less effective for attack duration and severity, with no consistent advantage on analgesic use or response; however, melatonin showed a more favourable tolerability profile, including lower risk of sleepiness (RR 0.49; 95% CI 0.28–0.87; *p* = 0.01).

**Conclusions:**

Melatonin demonstrates benefits over placebo for reducing migraine burden and improving patient-reported outcomes, with a favourable safety profile. While amitriptyline remains more potent for several efficacy endpoints, melatonin represents a reasonable preventive option, particularly as an adjunct during titration of first-line agents. Further head-to-head trials with standardised dosing and longer follow-up are warranted.

**Supplementary Information:**

The online version contains supplementary material available at 10.1007/s11916-025-01461-5.

## Introduction

Migraine is a chronic, inherited brain disorder that affects over a billion people of various socioeconomic statuses from all over the world [1]. Migraine is associated with both genetic and environmental factors and is best considered a neurovascular disorder although the pathophysiology of migraine is not fully elucidated. Migraine is a cyclical brain disorder consisting of four phases (interictal, prodrome, headache, postdrome) and diagnosed by the presence of moderate to severe headache, gastrointestinal symptoms, and hypersensitivity to sensory stimulation.

In a recent meta-analysis, it was estimated that half of individuals with migraine display a circadian pattern of attacks [2]. Moreover, migraine is strongly associated with disturbances in sleep, core clock genes, and urinary melatonin levels are low between and during attacks. Melatonin (N-acetyl-5-methoxytryptamine) is a chemical molecule present in practically all living creatures. Melatonin is produced in the pineal gland and then released into the circulation [3]. It is linked to circadian rhythm, seasonal responses, immunological function, antioxidant activity, free radical scavenging, and antinociception [3]. Melatonin has been shown to have antioxidant, anxiolytic, and analgesic, including opioid analgesia potentiation, antihypertensive, anti-inflammatory, and oncostatin properties [4]. Melatonin can protect the brain against direct toxic chemical damage by inhibiting free radicals and inflammatory factor production and there is increased evidence that supplementation of melatonin has efficacy for the preventive treatment of migraine [5].

Recent research suggests there may be multiple ways that melatonin may help in the prophylaxis of migraine [4]. The receptors for melatonin, MT1, and MT2, are located on the suprachiasmatic nucleus in the hypothalamus; importantly, the hypothalamus is thought to be a key driver in the initiation of migraine attacks and is involved in the process of chronic migraine [6, 7]. Melatonin is associated with the regulation of neurotransmitters and neurological pathways, reducing dopamine release, restricting nitric oxide synthesis, and antagonizing glutamate-induced excitotoxicity, among others [8]. Calcitonin gene-related peptide (CGRP) is a potent vasodilator whose release from the trigeminovascular system increases considerably during migraine attacks, resulting in vasodilation of brain blood vessels, inflammation, and pain signaling [9]. Melatonin can decrease CGRP release, regulating blood flow in the brain. Furthermore, melatonin is regarded as a potent analgesic with a powerful pain-relieving impact in pain disorders. In addition, the analgesic process involves increased β-endorphin release, activation of melatonin receptors, and augmentation of the brain’s γ-aminobutyric acidergic system [10].

A previous systematic review has suggested that melatonin may reduce the frequency of migraine attacks. However, they included only three studies in their analysis, which hindered any meaningful interpretation of data. Accordingly, we conducted a systematic review and meta-analysis to evaluate the efficacy (attack frequency, duration, severity, analgesic use.etc) and safety of melatonin for migraine prophylaxis, comparing it with placebo and other classical prophylactic drugs [5].

## Methods

This meta-analysis was registered in the International Prospective Register of Systematic Reviews (PROSPERO) under the registration number CRD42024593012. The study adhered meticulously to the Preferred Reporting Items for Systematic Reviews and Meta-Analyses (PRISMA) guidelines and the Cochrane Handbook [11, 12].

### Literature Search

On September 29, 2024, we performed a comprehensive search using PubMed, Cochrane, Scopus, Embase, and Web of Science databases. The search strategy included keywords such as (Melatonin OR Agomelatine OR Valdoxan OR Ramelteon OR Tasimelteon OR Melaxen OR Thymanax OR Circadin OR “Sleep hormone” OR Rozerem OR Hetlioz OR Slenyto OR PedPRM OR Epithalon OR Pinealtonin OR Melatonex OR Melatonina OR Melatonine OR Melatonon) AND (Migraine OR “Migraine headache “OR “Migraine Disorder” OR “Status Migrainosus” OR Headache).

### Eligibility Criteria and Study Selection

Eligible studies included randomised controlled trials (RCTs) that assessed the safety and efficacy of melatonin in the prevention and prophylaxis of individuals with migraine (both adult and pediatric). We used Rayyan.ai to manage studies from all databases, eliminating duplicate records [13]. Two authors (S.A, E.M.A) independently assessed titles, abstracts, and keywords for eligibility. Eligible studies underwent full-text assessment. If the full text was unavailable, attempts were made to contact authors via email for additional information; otherwise, the study was excluded. Discrepancies were resolved through consensus or consultation with a third reviewer. We excluded observational studies, case reports, case series, conference abstracts, unpublished articles, inaccessible full text despite attempts to acquire additional information from authors, any studies that were not published in English, and animal studies.

### Quality Assessment

The RoB 2 (Risk of Bias) tool [14] by Cochrane was used for quality assessment. It evaluates the quality of randomised trials based on specific domains such as randomisation process, adherence to intended intervention, outcome measurement, presence of missing outcome data, and selection of reported results. For each domain, we categorised the risk as “low risk,” “some concerns,” or “high risk”. Any discrepancies were resolved through discussion or, if necessary, by involving a third reviewer.

### Data Extraction

Two authors (S.A, E.M.A) used a standardised method to extract data, systematically recording relevant information in a predefined Excel sheet. This sheet included study characteristics such as study type, country, study duration, total number of patients, main outcomes, inclusion criteria, and conclusions. Additionally, patient demographics were captured, including age, gender, migraine attack duration, frequency, severity, and number of analgesics. The response rate was defined as the number of patients with more than a 50% decrease in monthly headache frequency during the follow-up period. The severity of the attack was measured by a visual analogue score (VAS). For the change calculation, we used a conservative correlation coefficient of 0.5, as recommended by the Cochrane review guidelines. Any discrepancies were resolved through consensus or by consulting the senior author.

### Data Synthesis and Heterogeneity Assessment

Our primary outcomes change from baseline to last follow-up include migraine attack duration in hours, migraine headache days, migraine monthly attack frequency, and the number of analgesic medications used during a migraine attack. Our secondary outcomes included headache severity (Visual Analog Scale), response rate (≥ 50% reduction in monthly headache frequency), sleep quality (Pittsburgh Sleep Quality Index, PSQI), disability (Migraine Disability Assessment, MIDAS; Pediatric MIDAS where applicable), and safety/adverse events (sleepiness, fatigue, dry mouth, dizziness, constipation, and worsening headache). We used R version 4.2.2 (2022-10-31) and RStudio version 2022.07.2 (2009–2022) (RStudio, Inc.) [15, 16]. For dichotomous outcomes, we calculated relative risk (RR) with the corresponding 95% confidence interval (CI), while mean differences (MD) were computed for continuous outcomes with CI. Statistical significance was determined at P values < 0.05. Heterogeneity was assessed based on p-value and I2. It was declared significant at a P value < 0.1. I2 was used to quantify the amount of heterogeneity present. When I < 50%, a fixed-effects model was selected. When I > 50%, a random-effects model was selected. If there is significant heterogeneity, it will be treated statistically with sensitivity analysis to assess the source of heterogeneity through study design. Subgrouping by population (adult vs. children) and type (episodic vs. chronic vs. unspecified) was done for efficacy outcomes whenever possible, and the primary analysis included at least three studies. Regarding the use of a funnel plot to assess the publication bias, according to Egger et al., the assessment was not statistically possible because it required a minimum of ten studies [17].

## Results

### Literature Search Results

**Fig. 1:**
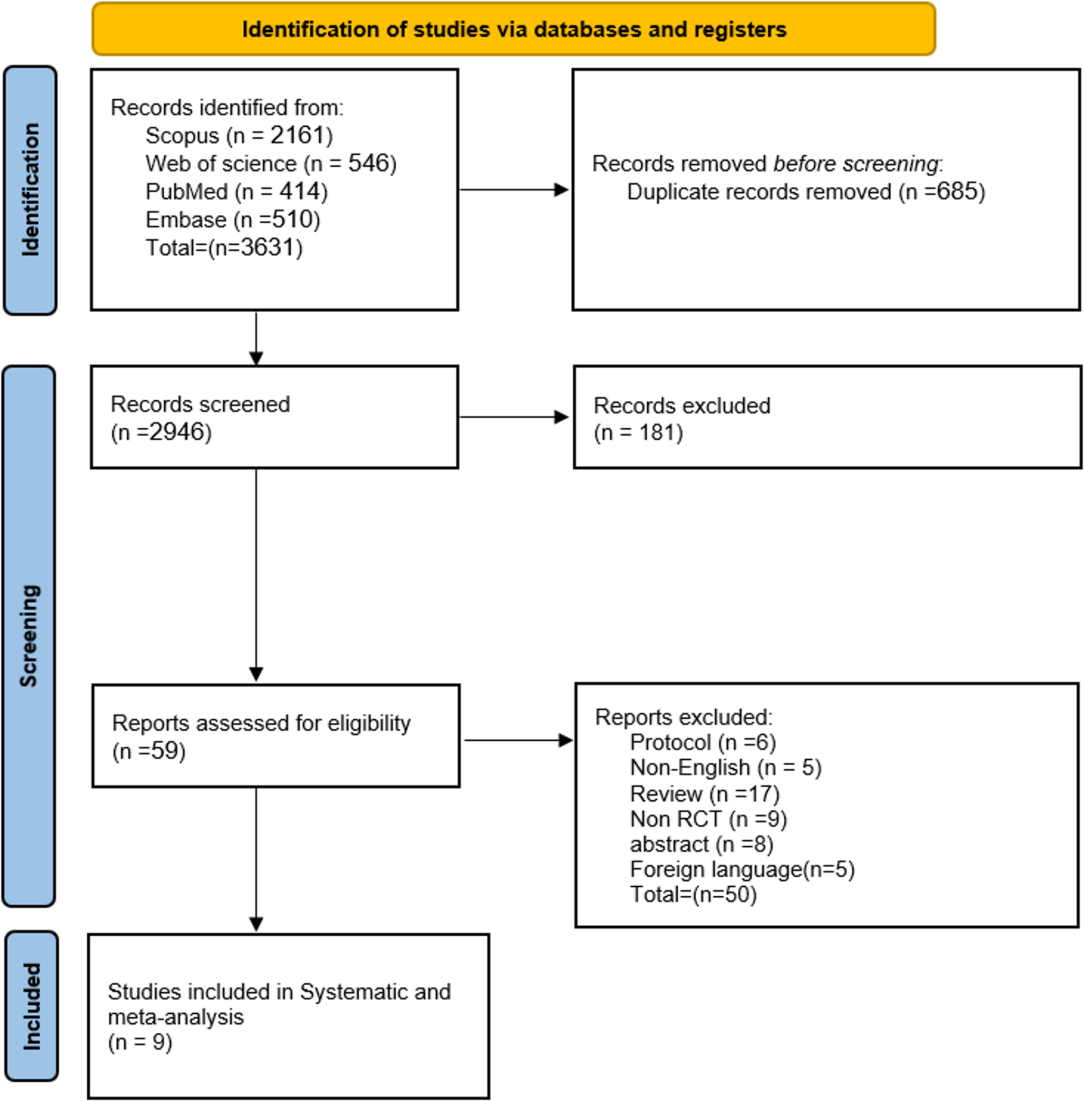
Prisma flow diagram

Following our comprehensive search, we initially identified 3631 records. After eliminating duplicates, 2,946 records were available for title and abstract screening. Subsequently, 59 articles appeared suitable for full-text screening. Ultimately, we included 9 RCTs in our meta-analysis [18–26]. The PRISMA flow diagram, depicted in Fig. 1, outlines this process.

### Characteristics of Individual Studies

**Fig. 2:**
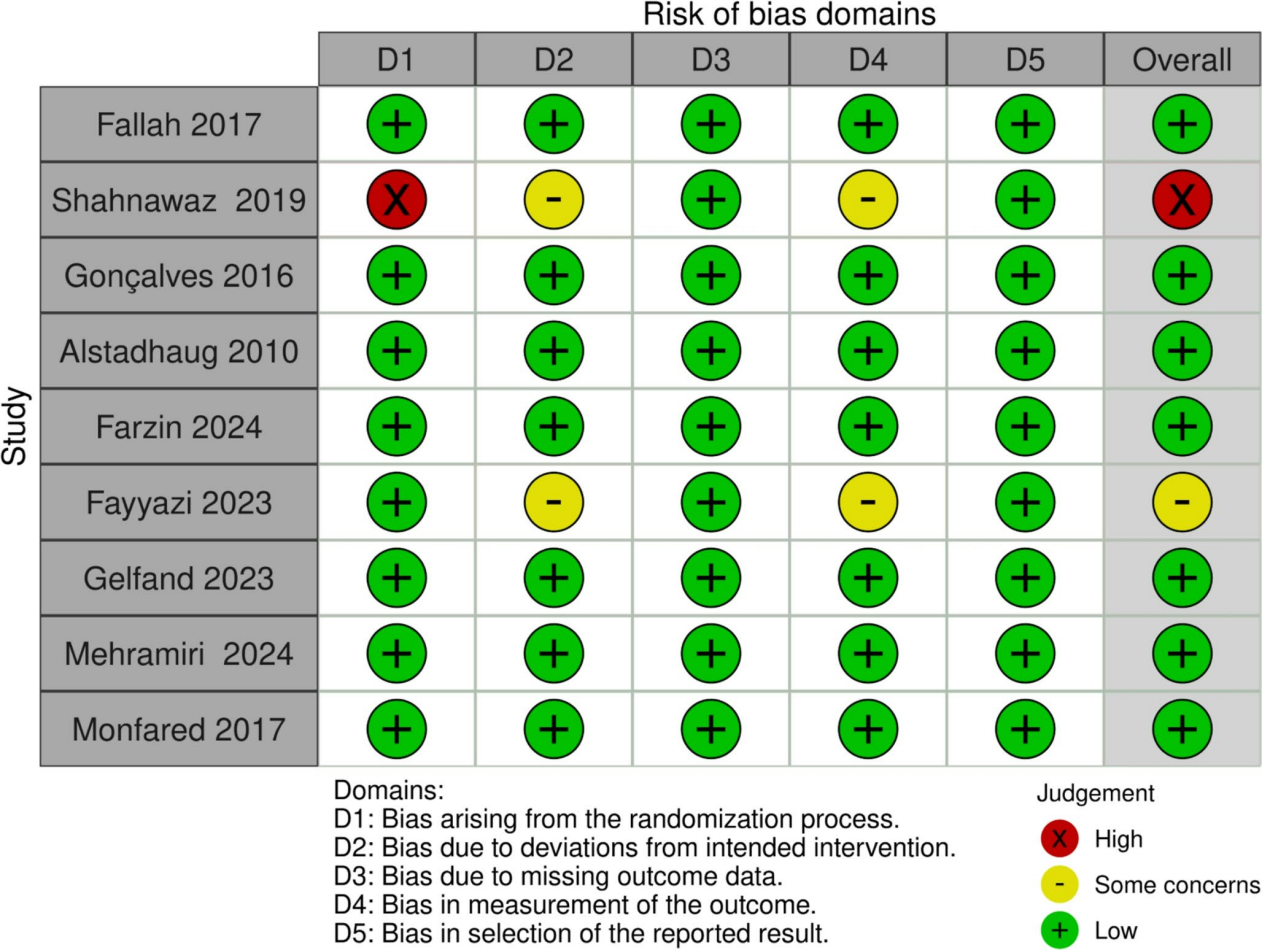
Shows the risk of bias assessment of the included study

A total of nine studies with 788 patients met the eligibility criteria and were included with a mean of 26.07 ± 15.36 years, 332 (42%) males, and melatonin doses ranges from 2 mg up to 6mg. We summarized the included studies and their patients’ baseline characteristics and the quality assessment in (Tables 1 and 2). respectively. The risk of bias 2 tool determined that the included seven RCTs showed a low risk of bias, one with high risk, and one with some Concerns (Fig. 2 and Supplementary Figure 1).

**Table 1:** Summary of the studies included

Study ID	Country	Study design	Study period	Sample size	Melatonin doses	Migraine Type	Inclusion criteria	Conclusion
Fallah 2017 [19]	Iran	RCT	8 months	80	Melatonin 0.3 mg/kg (Maximum 6 mg)	Migraine with aura and Migraine without aura	1- Age: children aged 5 to 15 years. 2- Migraine Diagnosis: Having migraine headaches (with or without aura) based on the second edition of the International Classification of Headache Disorders criteria, evaluated by a pediatric neurologist.	Amitriptyline and melatonin are both effective and safe options for pediatric migraine prevention, with amitriptyline showing greater efficacy.
Shahnawaz 2019 [20]	Pakistan	RCT	6 months	90	Melatonin 0.3 mg/kg (Maximum 6 mg)	Migraine with or without aura,	Patients exhibiting > 1 headache attack weekly, stating moderate or severe headache disability pedMIDAS > 20, and no history of using migraine prevention therapies	Both melatonin and amitriptyline demonstrate positive outcomes in children for migraine prophylaxis, with minimal and manageable side effects
Gonçalves 2016 [21]	Brazil	RCT	16 weeks (4 weeks baseline + 12 weeks treatment).	178	Melatonin 3 mg	Migraine with aura and Migrainewithout aura	1- Age: 18–65 years. 2- Migraine Diagnosis: migraine with or without aura, based on the International Classification of Headache Disorders, third edition (ICHD-3 β-version). 3- Duration of Migraine: migraine diagnosis for at least 1 year, with the onset before age 50.	Melatonin (3 mg) is superior to placebo for migraine prevention
Alstadhaug 2010 [22]	Norway	RCT (cross-over study)	6 months	48	Melatonin 2 mg	Migraine with aura and Migraine without aura	1- Age: Participants must be between 18 and 65 years old. 2- Migraine Diagnosis: Participants must have a diagnosis of migraine with or without aura for at least 1 year.	Prolonged-release melatonin (2 mg taken 1 h before bedtime) does not significantly outperform placebo in migraine prophylaxis.
Farzin 2024 [23]	Iran	RCT	Enrollment Phase: Six months. Intervention Phase: Three months.	100	Agomelatine 25 mg	Episodic migraine headaches without aura	Patients aged 18 to 60 years. Diagnosed with episodic migraine headaches without aura. Not receiving preventive treatment prior to the study.	No significant differences were observed between groups in headache frequency, monthly migraine days, severity, or disability indices during the study
Fayyazi 2023[24]	Iran	RCT	12 months	55	Melatonin 3 mg	Migraine without aura	1- Age: Children aged five to 15 years. 2- Diagnosis: Diagnosed with migraine according to the International Classification of Headache Disorders (ICHD-3 beta).	Adding melatonin to migraine treatment in children without sleep disorders significantly reduces headache frequency and improves treatment satisfaction
Gelfand 2023 [25]	USA	RCT	26 months	72	Melatonin 3 mg or 6 mg or (3 mg and 6 mg)	Migraine in children and adolescents	1- Age: 10–17 years. 2- Weight: ≥ 40 kg. 3- Migraine Diagnosis: Meets International Classification of Headache Disorders, 3rd edition (ICHD-3β) criteria for migraine in children and adolescents.	Melatonin was well tolerated with no serious adverse events.
Mehramiri 2024 [26]	Iran	RCT	4 months	60	Melatonin 3 mg	Chronic migraine	Patients with confirmed chronic migraine based on the criteria of the International Headache Society (IHS) by ICHD-3 diagnostic criteria, no primary internal and neurological complaints and no other reason for their headache, at least one year of migraine history	Melatonin proved more effective than placebo in reducing migraine attack frequency and duration, equally safe, and potentially beneficial for episodic migraine prevention in adults.
Monfared 2017 [27]	Iran	RCT	15 months	105	Melatonin 3 mg	Chronic migraine with and without aura	(1) Patients with chronic migraine who are capable of differentiating migraine from nonmigraine headaches (2) patients with no other reason for headache according to primary internal and neurological examinations	Melatonin as an adjuvant treatment was as effective as sodium valproate in chronic migraine prophylaxis but had better tolerability

RCT (Randomized Controlled Trial), PedMIDAS (Pediatric Migraine Disability Assessment Scale), ICHD-3 β (International Classification of Headache Disorders, 3rd edition, beta version), MMD (Monthly Migraine Days), MIDAS (Migraine Disability Assessment), IHS (International Headache Society), mg (Milligrams), mg/kg (Milligrams per kilogram), t (Test statistic), df (Degrees of freedom), p (p-value), USA (United States of America), Iran (Islamic Republic of Iran), KSA (Kingdom of Saudi Arabia), and ICHD-3 (International Classification of Headache Disorders, 3rd edition).

**Table 2:** Table of baseline characteristics

Study ID	Arms	Number of patients	Age (years) mean ± SD	Gender, *n* (%) Male	Mean attack duration (hours), Mean ± SD	Number of migraine headache Frequency, mean ± SD	Number of migraine headache days, mean ± SD	Mean headache Severity, mean ± SD	Mean migraine disability assessment (MIDAS) or (PedMIDAS), mean ± SD	Number of analgesics mean ± SD
Fallah 2017 [19]	Melatonin	40	10.57 ± 2.44	18(45)	2.06 ± 1.18	16.7 ± 6.68	NR	6.05 ± 1.63	PedMIDAS = 33.13 ± 9.17	12.32 ± 3.9
	Amitriptyline	40	10.11 ± 2.13	21 (52)	2.55 ± 1.85	15.8 ± 8.49	NR	6.41 ± 1.67	PedMIDAS = 31.4 ± 9.33	13.24 ± 2.6
Shahnawaz 2019 [20]	Melatonin	45	9.78 ± 2.3	25 (55)	2.34 ± 1.3	18.47 ± 5.8	NR	7.12 ± 1.9	PedMIDAS = 38.47 ± 11.9	NR
	Amitriptyline	45	9.48 ± 2.7	22 (48.8)	2.49 ± 1.5	17.24 ± 6.4	NR	7.65 ± 1.5	PedMIDAS = 41.68 ± 13.4	NR
Gonçalves 2016 [21]	Melatonin	60	36.9 ± 10.1	16 (26.7)	18.1 ± 13.8	NR	7.3 (2.8)	7.1 ± 1.9	NR	4.5 ± 1.9
	Amitriptyline	59	37.2 ± 11.2	15 (25.4)	16.7 ± 12.0	NR	7.2 (2.5)	7.0 ± 2.1	NR	4.6 ± 1.7
	Placebo	59	36.6 ± 13.7	14(23.7)	18.7 ± 17.3	NR	7.3 (3.1)	6.6 ± 1.4	NR	NR
Alstadhaug 2010 [22]	Migraine with or without aura	8	42.3 ± 10.8	2 (25)	NR	3.9 ± 1.4	NR	NR	NR	NR
	Migraine without aura	25	42.8 ± 9.3	5 (20)	NR	4.2 ± 1.3	NR	NR	NR	NR
	migraine with aura	13	39.8 ± 8.1	0	NR	4.3 ± 1.1	NR	NR	NR	NR
Farzin 2024 [23]	Agomelatine	50	18 to 60	39 (78)	NR	6.16 ± 1.65	NR	7.43 ± 1.1	MIDAS = 19.06 ± 5.31	NR
	Placebo	50		36 (72.0)	NR	11.94 ± 3.54	NR	7.04 ± 1.07	MIDAS = 17.47 ± 3.85	NR
Fayyazi 2023 [24]	Melatonin and Propranolol	28	10.6 ± 2.1	14(50)	NR	8.8 ± 4.6	NR	NR	PedMIDAS = 51.96 ± 44.19	NR
	Propranolol	27	9.6 ± 2	14(52)	NR	10.1 ± 7.2	NR	NR	PedMIDAS = 59.55 ± 56.53	NR
Gelfand 2023 [25]	Melatonin 6 mg	14	14.3 ± 2.4	2 (14)	NR	NR	NR	NR	NR	NR
	Melatonin 3 mg	14	15.2 ± 2.1	4 (29)	NR	NR	NR	NR	NR	NR
	Combined Melatonin groups	28	14.7 ± 2.2	6 (21)	NR	NR	NR	NR	NR	NR
	Placebo	14	15.4 ± 1.5	3 (21)	NR	NR	NR	NR	NR	NR
Mehramiri 2024 [26]	Melatonin	30	33.77 ± 6.89	11 (36.7)	NR	NR	NR	NR	MIDAS = 16.10 ± 3.59	NR
	Placebo	30	31.80 ± 8.67	14 (46.7)	NR	NR	NR	NR	MIDAS = 14.98 ± 3.78	NR
Monfared 2017 [27]	Melatonin	35	38.9 ± 9.2	51 (48.57)	19.8 ± 19.8	NR	NR	7.3 ± 1.2	MIDAS = 15.2 ± 7.2	7.3 ± 2.1
	Valproic acid	35			19.5 ± 18.1	NR	NR	7.4 ± 1.8	MIDAS = 16.1 ± 4.1	7.4 ± 3.5
	Placebo	35			19.6 ± 18	NR	NR	7.3 ± 1.1	MIDAS = 16 ± 3.2	7.2 ± 3.8

NR (Not Reported), MIDAS (Migraine Disability Assessment Scale), PedMIDAS (Pediatric Migraine Disability Assessment Scale), SD (Standard Deviation), and n (number of patients).

### Result of Meta-analysis

#### Migraine Attack Duration in Hours

**Fig. 3:**
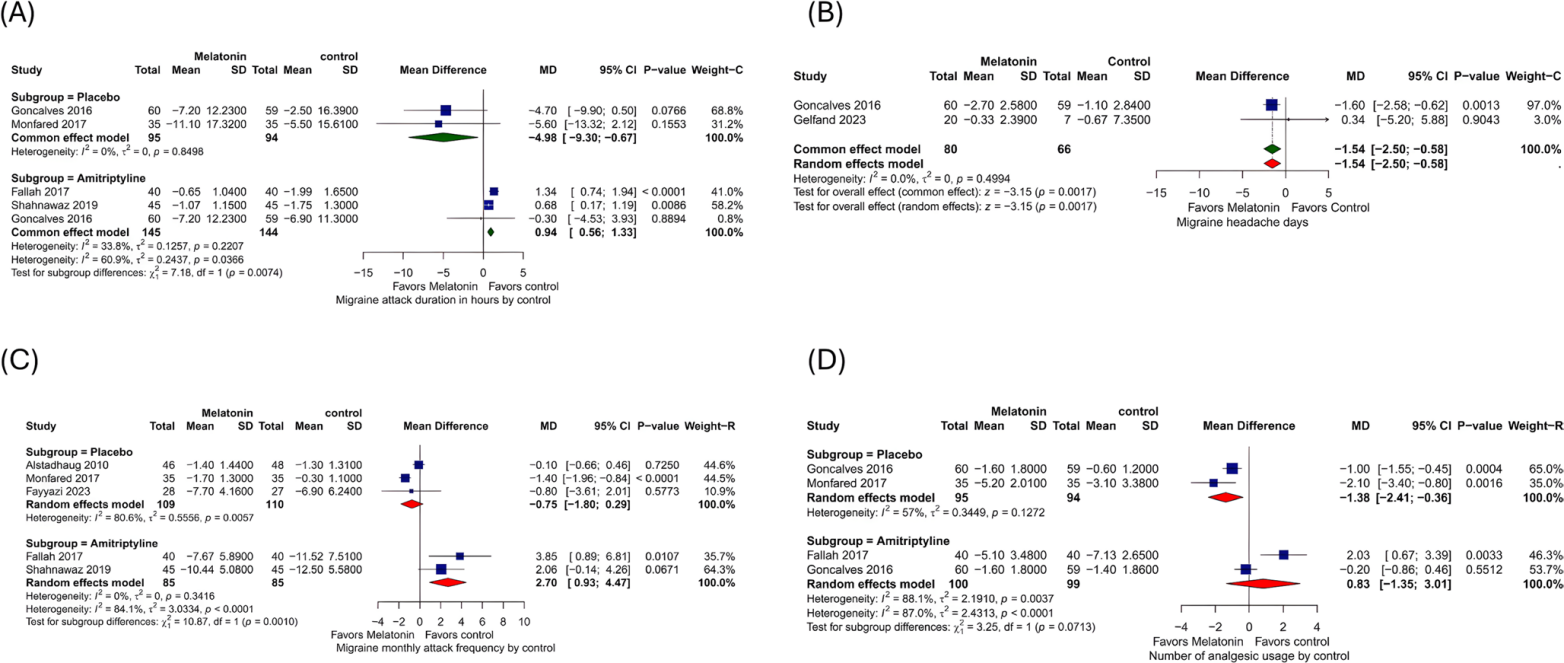
Forest plots show cc migraine attack duration in hours, (**B**) Migraine headache days, (**C**) Migraine monthly attack frequency, and (**D**) Number of analgesic usage

The comparison between melatonin and placebo shows that two studies have reported on the duration of migraine attacks. Melatonin significantly decreases attack duration by 4.98 h compared to placebo (MD = −4.98, 95% CI: [−9.3, −0.67], *p* = 0.02). There was no heterogeneity (I² = 0%, *p* = 0.85). The analysis of 3 studies comparing melatonin vs. amitriptyline shows that amitriptyline significantly decreases attack duration by 0.94 h compared to melatonin (MD = 0.94, 95% CI: [0.56, 1.33], *p* < 0.01). There was no heterogeneity (I² = 34%, *p* = 0.22). (Fig. 3A). Subgroup analysis by age of population showed a significant reduction in migraine attack duration, favoring amitriptyline in children (MD = 0.95 [95% CI: 0.56 to 1.34], *p* < 0.0001, I² = 62.8%, *p* = 0.1). In comparison, no significant difference was observed between melatonin and amitriptyline in adults (MD = −0.30 [95% CI: −4.53 to 3.93], *p* = 0.8894, I² = 60.9%) (Supplementary Fig. 2). Subgroup analysis by type of headache could not provide a clear conclusion regarding migraine subtype, as only the subgroup with non-specified migraine (with or without aura) showed a statistically significant reduction in migraine duration, favoring amitriptyline (MD = 0.95 [95% CI: 0.56 to 1.34], *p* < 0.0001, I² = 62.8%). Other subgroups, including episodic and chronic migraine, did not show significant differences between melatonin and placebo. However, there was a favorable trend toward melatonin (MD = −4.70 [95% CI: −9.90 to 0.50] for episodic migraine, and − 5.60 [95% CI: −13.32 to 2.12] for chronic migraine) (supplementary Fig. 3).

#### Migraine Headache Days

A comparison of melatonin vs. placebo shows that two studies reported migraine headache day frequency. Melatonin significantly decreases headache days by a mean difference of 1.54 days compared to placebo (MD = −1.54 days, 95% CI: [−2.50, −0.58], *p* < 0.01), and there was no heterogeneity (I² = 0%, *p* = 0.5). (Fig. 3B).

#### Migraine Monthly Attack Frequency

The melatonin vs. placebo group shows that three studies reported on the migraine monthly attack frequency. The overall effect was (MD = −0.75, 95% CI: [−1.80, 0.29], *p* = 0.16). There was Considerable heterogeneity (I² = 80%, *p* = 0.006). After excluding Alstadhaug 2010 from the analysis, melatonin significantly decreased monthly attack frequency by 1.38 attacks compared to placebo (MD = −1.38, 95% CI: [−1.93, −0.83], *p* < 0.01), and there was no heterogeneity (I² = 0%) (Fig. 3C). (Supplementary Fig. 4).

#### Number of Analgesic Usages

Melatonin vs. placebo comparison Shows that two studies reported the number of analgesic usages for acute attacks. Melatonin significantly decreases the number of analgesic usages by 1.38 compared to placebo (MD = −1.38, 95% CI: [−2.41, −0.36], *p* < 0.01) and there was no significant heterogeneity (I² = 57%, *p* = 0.13). While the analysis of 2 studies comparing melatonin vs. amitriptyline shows that the overall effect was (MD = 0.83, 95%). CI: [−1.35, 3.01], *p* = 0.45). There was substantial heterogeneity (I² = 88%, *p* < 0.01). (Fig. 3D).

#### Migraine Severity

**Fig. 4:**
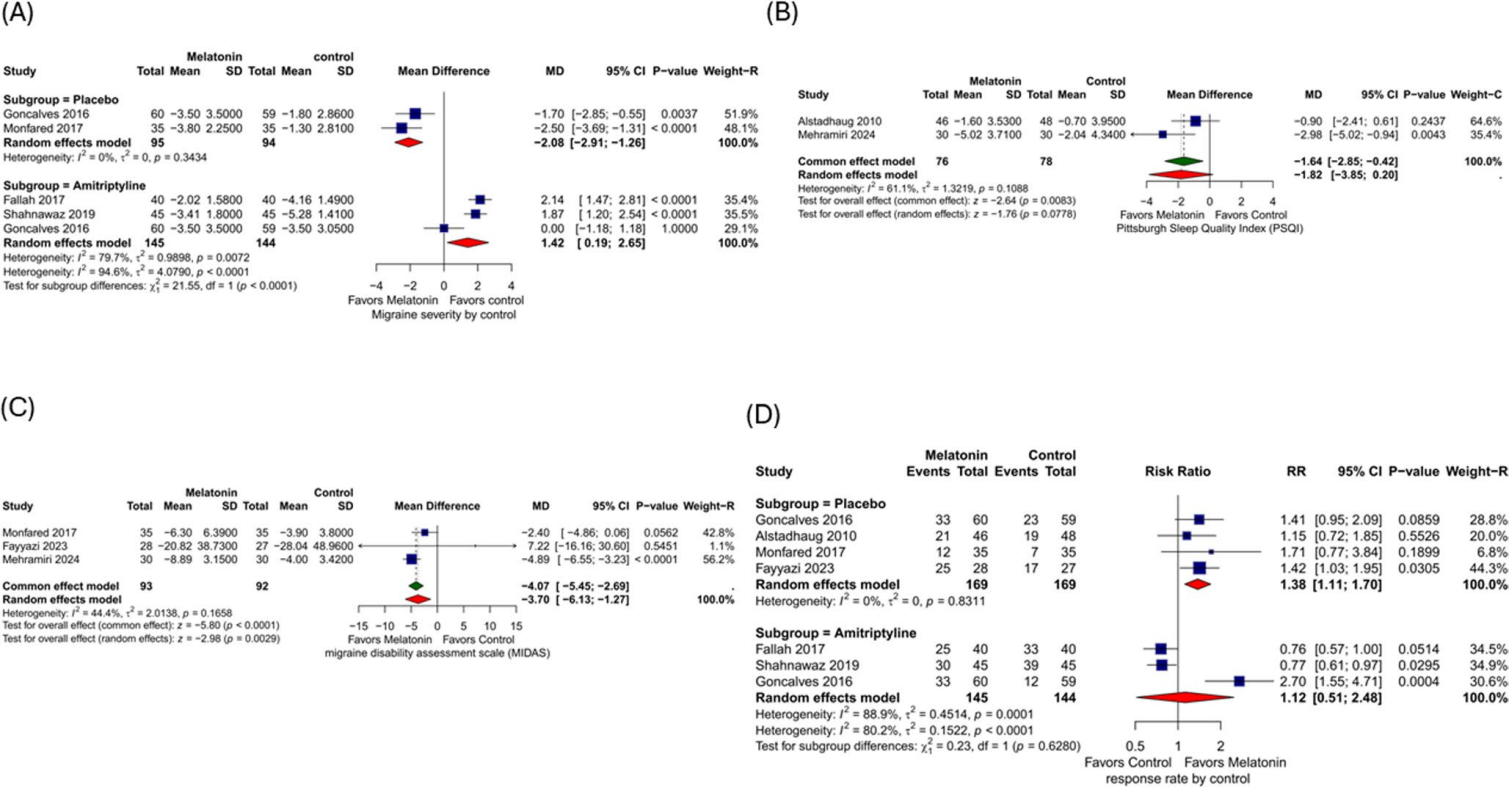
(**A**) Migraine severity, (**B**) Pittsburgh Sleep Quality Index (PSQI), (**C**) Migraine disability assessment scale (MIDAS), and (**D**) Response rate

A Melatonin vs. placebo comparison shows that two studies reported on migraine severity. Melatonin significantly decreases migraine severity by 2.08 compared to placebo (MD = −2.08, 95% CI: [−2.91, −1.26], *p* < 0.01). There was no heterogeneity (I² = 0%, *p* = 0.34). ). The analysis of three studies comparing melatonin vs. amitriptyline shows that the overall effect was MD = 1.42 (95% CI: [0.19, 2.65], *p* = 0.02). There was substantial heterogeneity (I² = 80%, *p* < 0.01) (Fig. 4A). After leaving out Gonçalves 2016. Amitriptyline significantly reduces migraine severity by 2 points compared with melatonin (MD = 2, CI: [1.53, 2.48], *p* < 0.01). There was no heterogeneity (I² = 0%) (Supplementary Fig. 5). Subgroup analysis demonstrated that this significant effect against placebo persisted in both the episodic and chorionic groups and didn’t differ by population (Supplementary Figs. 6 and 7).

#### Pittsburgh Sleep Quality Index (PSQI)

The melatonin vs. placebo comparison shows that two studies reported on PSQI. There was a statistically significant difference between the two interventions (MD = −1.64, 95%). CI: [−2.85, −0.42], *p* = 0.008. There was no significant heterogeneity (I² = 61%, *p* = 0.11). (Fig. 4B)

#### Migraine Disability Assessment (MIDAS)

The melatonin vs. placebo comparison shows that three studies reported on MIDAS. The overall effect was (SMD= −4.07, 95% CI: [−5.45 - −2.69], *p* < 0.0001) (Fig. 4**C)**. There was moderate heterogeneity (I² = 44.4%, *p* = 0.16). When subgrouped by population, in adults, Melatonin significantly reduced migraine severity compared to placebo (MD = −4.11, CI: [−5.49, −2.73], *p* < 0.001). In children, the difference was not statistically significant (MD = 7.22, CI: [−16.16, 30.60], *p* = 0.55) -it was based on one outlier study, Fayyazi et al. (Supplementary Fig. 8). Similarly, subgrouping by headache type showed a favor of melatonin in chronic migraine (MD = −4.11, CI: [−5.49, −2.73], *p* < 0.001) (Supplementary Fig. 9).

#### Response Rate

A comparison of melatonin vs. placebo shows that four studies reported on the response rate. The overall effect shows that patients had a significantly higher response to melatonin than placebo (RR = 1.38, 95% CI: [1.11, 1.7], *p* < 0.01). There was no heterogeneity (I² = 0%, *p* = 0.83), (Fig. 4D.). Subgroup analysis by population also demonstrated a significant effect in both groups. In adults, melatonin increased the response rate by 1.34 times compared to placebo (RR = 1.34, 95% CI: [1.01, 1.78], *p* = 0.04), while in children (RR = 1.42 [95% CI: 1.03 to 1.95], *p* = 0.0305). (Supplementary Fig. 10)

The comparison of melatonin vs. amitriptyline shows that three studies reported response rates. The overall effect was not statistically significant (RR = 1.12, 95% CI: [0.51, 2.48], *p* = 0.77). There was substantial heterogeneity (I² = 89%, *p* < 0.01). After leaving Gonçalves 2016, the overall effect shows that amitriptyline increases the response rate by 24% compared with melatonin (RR = −0.76, 95% CI: [0.64, 0.92], *P* < 0.01). There was no heterogeneity (I² = 0%) (Fig. 4D, Supplementary Fig. 11). Compared with placebo in patients with episodic migraine, the effect showed a non-significant trend in favour of melatonin (RR = 1.30, 95% CI: [0.96, 1.76]) (Supplementary Fig. 12).

#### Safety Outcomes

**Fig. 5:**
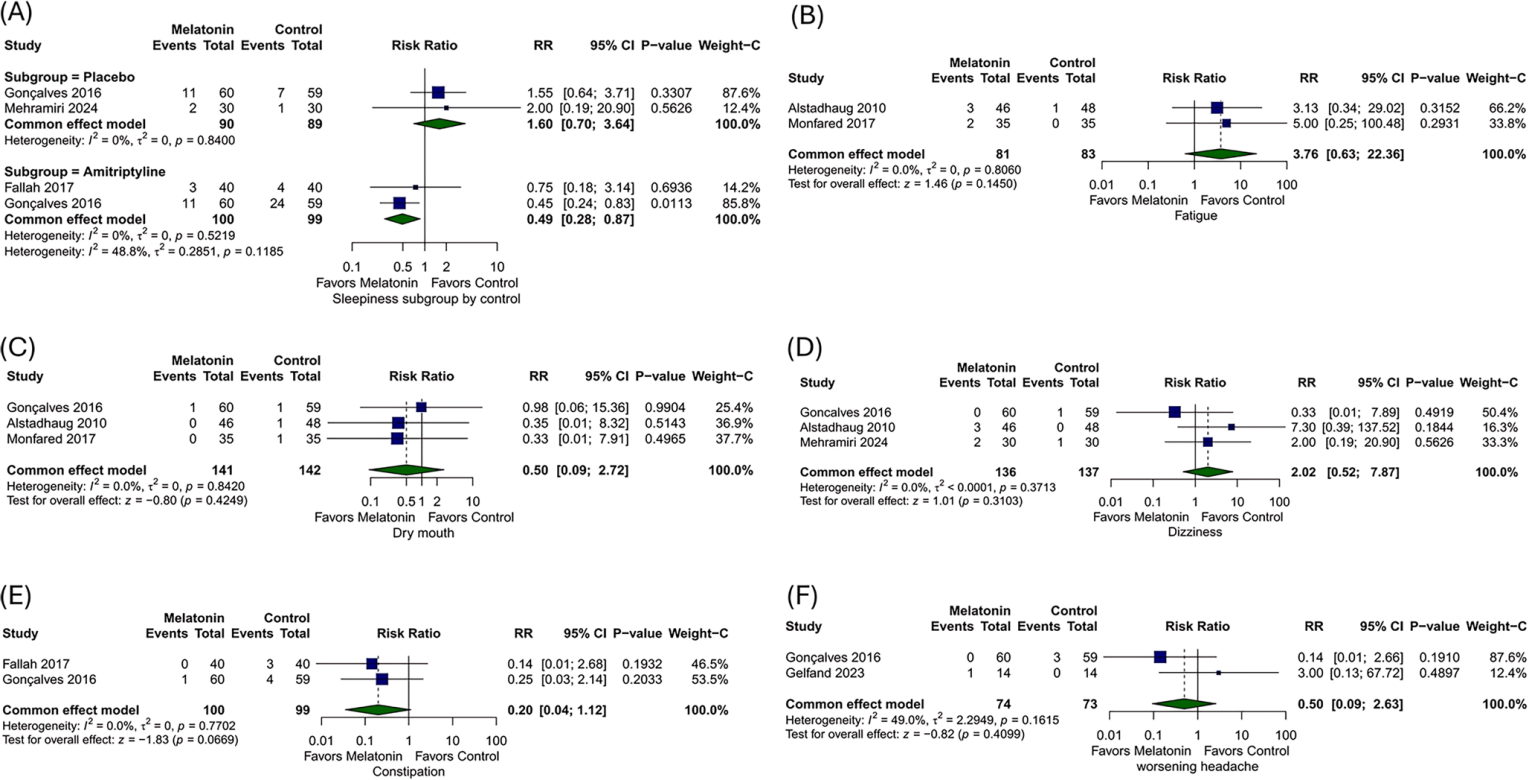
Forest plots for (**A**) Sleepiness, (**B**) Fatigue, (**C**) Dry mouth, (**D**) Dizziness, (**E**) Constipation and (**F**) worsening headache

The safety outcomes of melatonin were compared with placebo and amitriptyline for several adverse effects. For sleepiness, two studies comparing melatonin with placebo showed no statistically significant difference (RR = 1.6, 95% CI: [0.7, 3.64], *p* = 0.26, I² = 0%) (Fig. 5A). However, when comparing melatonin to amitriptyline, melatonin significantly reduced sleepiness by 51% (RR = 0.49, 95% CI: [0.28, 0.87], *p* = 0.01, I² = 0%) (Fig. 5A). For fatigue, two studies comparing melatonin with placebo found no statistically significant difference (RR = 3.76, 95% CI: [0.63, 22.36], *p* = 0.15, I² = 0%) (Fig. 5B). Similarly, dry mouth showed no significant difference between melatonin and placebo (RR = 0.50, 95% CI: [0.09, 2.72], *p* = 0.42, I² = 0%) (Fig. 5C).

For dizziness, three studies comparing melatonin with placebo found no statistically significant difference (RR = 2.02, 95% CI: [0.52, 7.87], *p* = 0.31, I² = 0%) (Fig. 5D). Finally, the comparison of constipation and worsening headaches between melatonin and amitriptyline showed no statistically significant difference (RR = 0.2, 95% CI: [0.04, 1.12], *p* = 0.07, I² = 0%) (Fig. 5E), (RR = 0.50, 95% CI: [0.09, 2.63], *p* = 0.41, I² = 49%) (Fig. 5F).

## Discussion

Our search yielded 9 articles for comparison, from 2010 to 2024. When compared to placebo, melatonin significantly improved several migraine-related outcomes. It reduced migraine attack duration by 4.98 h, decreased the number of migraine headache days by 1.89 days, and lowered headache severity by 2.52 points on the VAS scale. Furthermore, melatonin decreased the number of analgesic usages by 1.38, and patients receiving melatonin were 1.38 times more likely to experience a reduction in monthly headache frequency. Other outcomes, such as improvements in the PSQI and MIDAS, were also noted, though they did not reach statistical significance.

Compared with amitriptyline, melatonin was less effective in several areas. Migraine attack duration increased by 0.94 h with melatonin, and the frequency of migraine attacks rose by 2.7 per month. Migraine severity also increased by 2 points compared to amitriptyline. Additionally, melatonin did not significantly reduce analgesic use or improve response rates compared with amitriptyline. Despite these differences, melatonin had a better safety profile, with a 51% reduction in sleepiness. Other side effects, such as fatigue, dry mouth, dizziness, and constipation, were also evaluated but did not differ significantly between melatonin and other treatments. Although amitriptyline has been associated with weight gain [27], only the study by Goncalves et al. reported increased weight gain as a side effect in 5% of amitriptyline users compared to none in melatonin users.

In exploring between-study heterogeneity, three studies were especially sources of heterogeneity. Alstadhaug 2010, a small crossover trial using prolonged-release 2 mg, introduced clinical and design non-comparability (carryover risk, lower effective night-time peak), inflating heterogeneity in monthly attack frequency; excluding it reduced I² and yielded a directionally consistent, statistically significant pooled MD favouring melatonin vs. placebo. Gonçalves 2016, a larger three-arm adult RCT with amitriptyline 25 mg and melatonin 3 mg, was influential when pooled with pediatric trials for severity and response rate, likely due to age-mixing, dosing of the active comparator, and outcome ascertainment differences; its removal reduced I², clarified the expected superiority of amitriptyline on these endpoints without altering conclusions for melatonin vs. placebo. Notably, the non-significant PedMIDAS effect in the pediatric subgroup was based on one outlier pediatric study (Fayyazi et al.). Because this subgroup estimate rests on a single small trial with a differing clinical context, it should be considered hypothesis-generating rather than definitive.

The findings of this meta-analysis are consistent with prior studies. For instance, Mehramir et al. and Fayyazi et al. also found significant reductions in migraine frequency and attack duration with melatonin compared to placebo [23, 25]. Monfared et al. reported a reduction in analgesic use, aligning with the findings of this meta-analysis [26]. However, improvements in PSQI and MIDAS scores were not significant in our study, similar to Alstadhaug et al., who found no substantial improvement in sleep quality [21]. A previous meta-analysis by Puliappadamb et al. supports several of our findings, particularly regarding the effects of melatonin compared to a placebo [5]. Their study demonstrated that melatonin significantly reduced migraine attack duration by 5.02 h, reduced the frequency of migraine attacks by 1.00 days, and lowered migraine severity by 1.93 points on the VAS scale. Additionally, it showed a significant reduction in analgesic usage, with a mean decrease of 1.43 uses compared to placebo. However, when compared to standard therapies like amitriptyline, Puliappadamb et al., found no significant differences in migraine frequency, duration, severity, or analgesic use, aligning with their conclusion that melatonin was not superior to conventional treatments. Despite this, melatonin’s safety profile was favourable, with no significant difference in adverse drug reactions—such as drowsiness and fatigue -when compared to both placebo and standard treatments [5].

The Puliappadamb et al. meta-analysis had several notable limitations, especially when compared to our more comprehensive analysis [5]. First, their study only included three clinical trials with a total of 236 patients, which limits the generalizability of their findings. In contrast, our meta-analysis included nine RCTs with a total of 788 patients, providing a more robust data set and offering stronger, more reliable conclusions regarding melatonin’s efficacy and safety for migraine prophylaxis. Additionally, the smaller sample size in Puliappadamb et al.‘s study may have affected the statistical power of their results, especially when comparing melatonin to amitriptyline and placebo. By including more trials, our analysis provides a broader evaluation of melatonin across various patient subgroups, thus improving the external validity of our findings.

The greater efficacy of amitriptyline compared to melatonin in reducing migraine frequency and severity is likely due to its stronger modulation of neurotransmitters [28]. Melatonin’s benefits may derive from its regulatory effects on circadian rhythm and its anti-inflammatory properties [29]. Despite being less effective than amitriptyline, melatonin’s better safety profile may make it a favorable choice for patients sensitive to side effects such as sleepiness. The lack of significant improvements in sleep quality and MIDAS scores in this analysis, despite reductions in migraine frequency and severity, may be due to the high placebo response often seen in migraine studies, particularly in pediatric populations, as reported by Gelfand et al. [24].

We found only one study, Farzin et al., that was excluded from meta-analysis on the use of a melatonin agonist (agomelatine 25 mg nightly) in adults with episodic migraine without aura (parallel, triple-blind RCT; *n* = 100; 3-month follow-up, which further complements the efficacy of melatonin and opens the door for melatonin agonist use in future research [22]. Agomelatine, an MT1/MT2 agonist and 5-HT₂C antagonist, demonstrated statistically significant improvements versus control (vitamin B1) in monthly headache frequency (*p* = 0.009), monthly migraine days (*p* = 0.025), and within-group reductions in headache severity and MIDAS (both *p* < 0.001), consistent with a clinically meaningful preventive signal. However, because agomelatine’s serotonergic antidepressant activity could confound effect attribution relative to melatonin’s primarily circadian/anti-inflammatory mechanisms, and because the comparator and outcome ascertainment differ from several melatonin trials, we did not pool agomelatine with melatonin in our primary estimates [30]. Instead, we cite this study as supportive evidence that melatonergic modulation may benefit migraine prevention in selected adult populations.

### Strengths and Limitations

A major strength of this meta-analysis is the inclusion of only RCTs, which ensures high reliability and validity of the findings. By focusing on RCTs, the study minimizes bias and provides a stronger foundation for assessing melatonin’s efficacy and safety. Additionally, the meta-analysis evaluates a wide range of outcomes, including migraine duration, frequency, severity, analgesic use, and safety measures such as sleepiness, fatigue, and other side effects. This comprehensive approach offers a well-rounded understanding of melatonin’s impact on migraine management. Furthermore, the comparison of melatonin with both placebo and amitriptyline allows for a more detailed evaluation of its relative efficacy, demonstrating its benefits as a potential treatment option. The focus on safety is another strength, as the study highlights melatonin’s favorable safety profile, with fewer side effects compared to amitriptyline, which is important for patient compliance and long-term use.

However, the study does have some limitations. The relatively short follow-up periods across the included RCTs limit the ability to assess the long-term efficacy and safety of melatonin. Additionally, variations in melatonin dosage and formulation across trials introduce uncertainty about the optimal dosage for migraine prevention, reducing the generalizability of the findings. Another limitation is the differences in populations and migraine subtypes. However, we conducted subgroup analyses based on them and explained some of the original heterogeneity in the primary analyses. It is important to note that most of these subgroups included only a limited number of studies —1 or 2 at most —and their results cannot be indicative of differential effects of melatonin across populations or types. Another limitation is the high placebo response observed in several trials, which may have diminished the observable effect of melatonin, making it harder to detect its actual therapeutic impact. Lastly, some outcomes exhibited significant heterogeneity, particularly in comparisons involving amitriptyline. Although leave-one-out analyses and subgroup analyses were conducted to address this, the variability between studies still affects the consistency and interpretation of specific results.

### Implications and Future Directions

Melatonin may be a more suitable option for patients prioritising safety and tolerability, particularly those concerned about side effects like sleepiness. It significantly reduces migraine duration and headache days compared to placebo, making it a good alternative for patients with lower frequencies of migraine. However, for those requiring greater control over migraine attack frequency, severity, and duration, amitriptyline appears to be more effective despite its higher side effect burden. In summary, melatonin 3 mg immediate-release at bedtime may be considered as an adjunct during titration of established preventive medications, when prevention is warranted but disability is minimal, or as an alternative in patients intolerant to side effects.

Several gaps in the current literature warrant further investigation. First, the optimal dose and timing of melatonin for migraine prevention are still unclear, with varying results depending on dosage, as seen in studies by Mehramir (2024) and Alstadhaug (2010). Further research is needed to determine the most effective dosing regimen [21, 25]. Second, the long-term efficacy and safety of melatonin have not been extensively studied, with most trials focusing on short-term outcomes. Additionally, more research is needed to understand melatonin’s mechanisms of action, particularly its interactions with migraine pathophysiology and hormonal fluctuations, as suggested by Monfared et al. [26]. Fourth, the high placebo response, Gelfand et al., reported that placebo responses were almost equal to melatonin treatment in some cases [24]. Future studies should consider designs that can mitigate this effect, such as incorporating a placebo run-in phase, to better distinguish melatonin’s effects from placebo.

Additionally, comparative studies involving melatonin and other common migraine treatments, such as valproate, topiramate, and newer CGRP inhibitors, are needed to clarify their position within the migraine treatment hierarchy. While some studies, such as those by Shahnawaz and Monfared, found melatonin to be less effective than amitriptyline or valproate [19, 26], others suggested that melatonin offers similar efficacy with fewer side effects [20]. More standardized and head-to-head trials are necessary to conclusively determine melatonin’s role with these treatments.

Another knowledge gap is related to the patient population studied. While many studies have focused on pediatric and adolescent populations [20, 21], fewer have thoroughly examined the effects of melatonin in adults [25, 26]. Additionally, there is some indication that melatonin may work better for specific clinical presentations, such as those related to sleep disorders [23], but more research is required to clarify these distinctions and potentially guide personalized treatment approaches. Lastly, future research should investigate the potential for combination therapies involving melatonin. Preliminary evidence from electronic health record analysis of adults with insomnia has reported an association between melatonin use and higher subsequent rates of heart failure, heart failure hospitalization, and all-cause mortality [31]. Although these findings are observational and do not establish causality, they reinforce the need for long-term safety studies in migraine populations for insomnia and cardiometabolic comorbidity. Given its favorable safety profile, melatonin may serve as an adjunct to other migraine therapies, potentially allowing for lower doses, thereby potentially reducing side effects while maintaining efficacy. Studies exploring this synergistic potential could provide valuable insights for improving patient outcomes.

## Conclusion

In conclusion, while melatonin demonstrates moderate efficacy in reducing migraine symptoms compared to placebo, it is less effective than amitriptyline in reducing the frequency and severity of attacks. However, melatonin’s superior safety profile makes it a valuable option for patients who prioritize tolerability. Further research is needed to optimize dosing and to explore the long-term role, including safety and efficacy, of melatonin in migraine prevention.

## Supplementary Information

Below is the link to the electronic supplementary material.

Supplementary Material 1

## Data Availability

The data used in this study is available upon reasonable request.
