# Supplementary material for: Efficacy and Safety of Melatonin in Migraine Prophylaxis: A Systematic Review and Meta-Analysis of Randomized Controlled Trials
Source: Curr Pain Headache Rep. 2026 Feb 2;30(1):25. doi: 10.1007/s11916-025-01461-5 (PMC12864225; doi:10.1007/s11916-025-01461-5)

**List of Figures**

[**Supplementary Figure 1: Quality assessment of the included studies: For randomized controlled** 2](#_Toc212929019)

[**Supplementary Figure 2: Subgroup analysis by age — migraine attack duration** 3](#_Toc212929020)

[**Supplementary Figure 3: Subgroup analysis by headache type — migraine attack duration** 4](#_Toc212929021)

[**Supplementary Figure 4: Sensitivity analysis monthly migraine attack frequency**. 5](#_Toc212929022)

[**Supplementary Figure 5: Sensitivity analysis migraine severity.** 5](#_Toc212929023)

[***Supplementary Figure 6: Subgroup analysis by headache type — migraine severity.*** 5](#_Toc212929024)

[***Supplementary Figure 7: Subgroup analysis by population— migraine severity*** 6](#_Toc212929025)

[***Supplementary Figure 8: Subgroup analysis by population — migraine disability (MIDAS).*** 7](#_Toc212929026)

[***Supplementary Figure 9: Subgroup analysis by headache type - migraine disability (MIDAS)*** 8](#_Toc212929027)

[***Supplementary Figure 10: Subgroup analysis by population — response rate*** 9](#_Toc212929028)

[***Supplementary Figure 11: Sensitivity analysis —response rate.*** 10](#_Toc212929029)

[***Supplementary Figure 12: Subgroup analysis by headache type — response rate.*** 11](#_Toc212929030)

**Supplementary Figure 1: Quality assessment of the included studies: For randomized controlled**

*
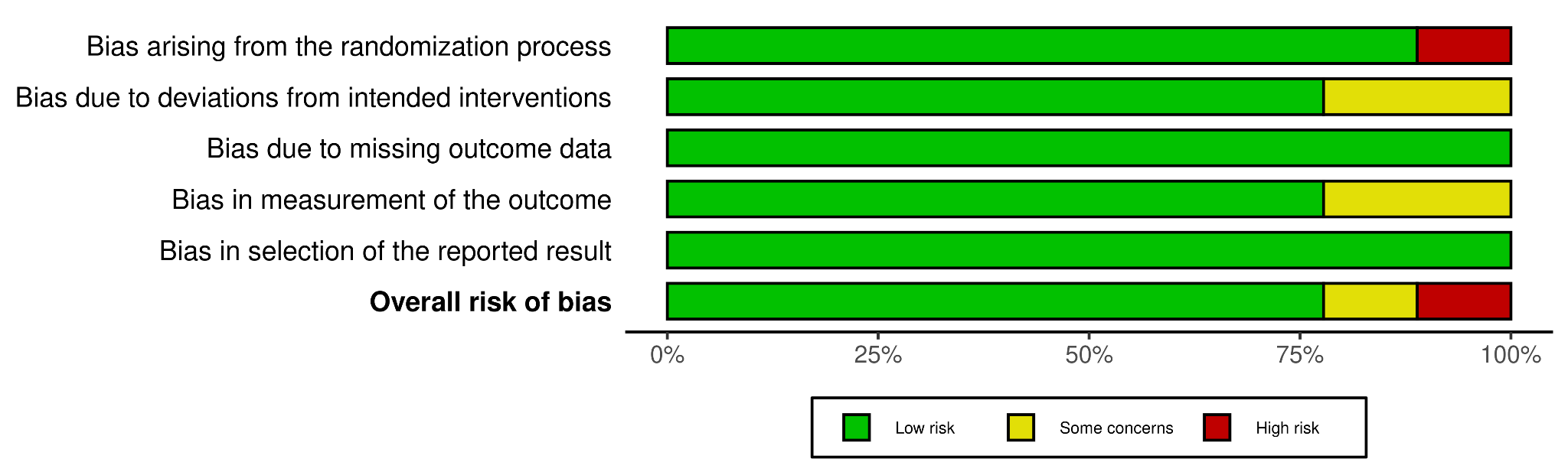
*

*:*

**Supplementary Figure 2: Subgroup analysis by age — migraine attack duration**

**
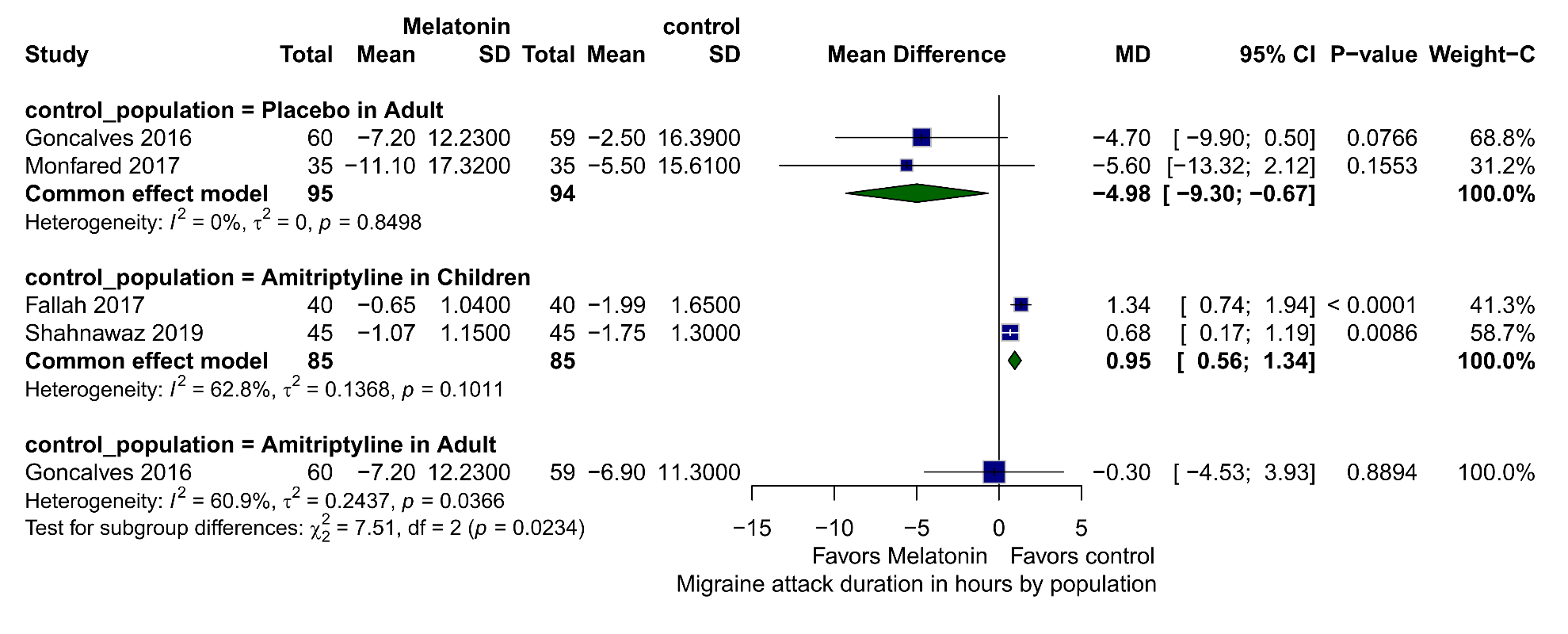
**

**Supplementary Figure 3: Subgroup analysis by headache type — migraine attack duration**


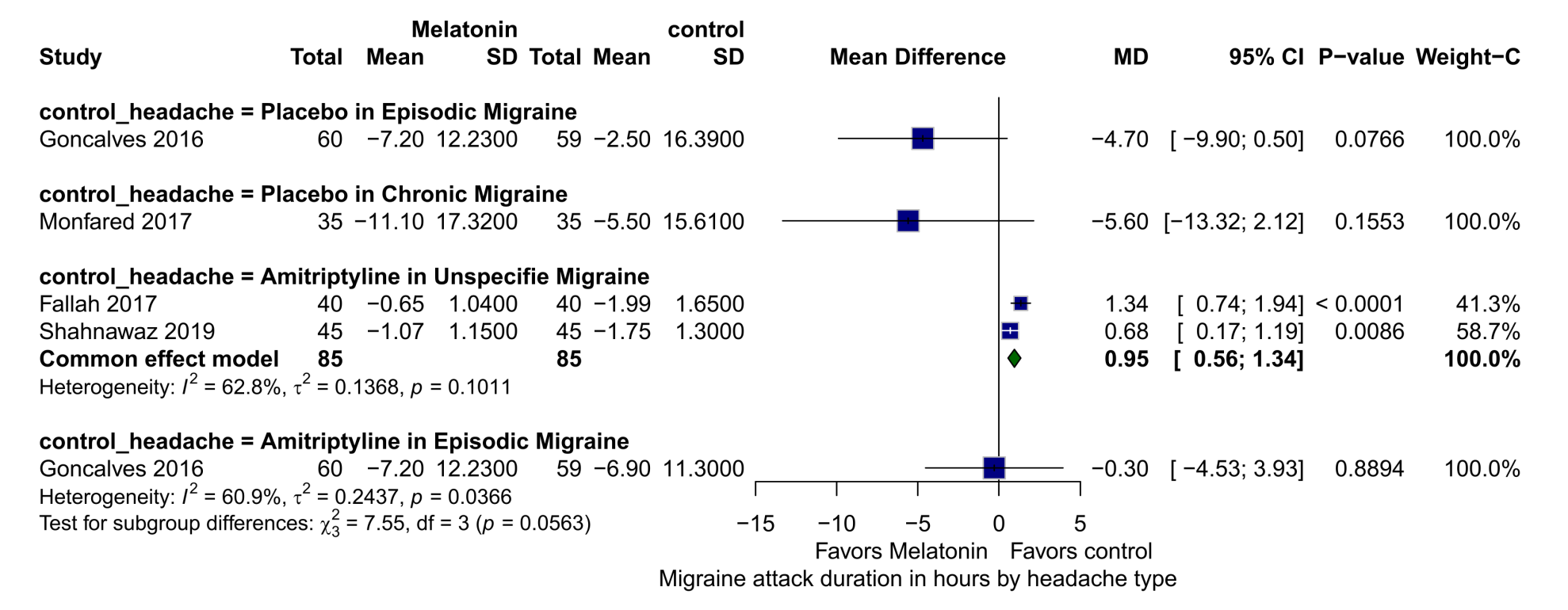


**Supplementary Figure 4: Sensitivity analysis monthly migraine attack frequency**.

***
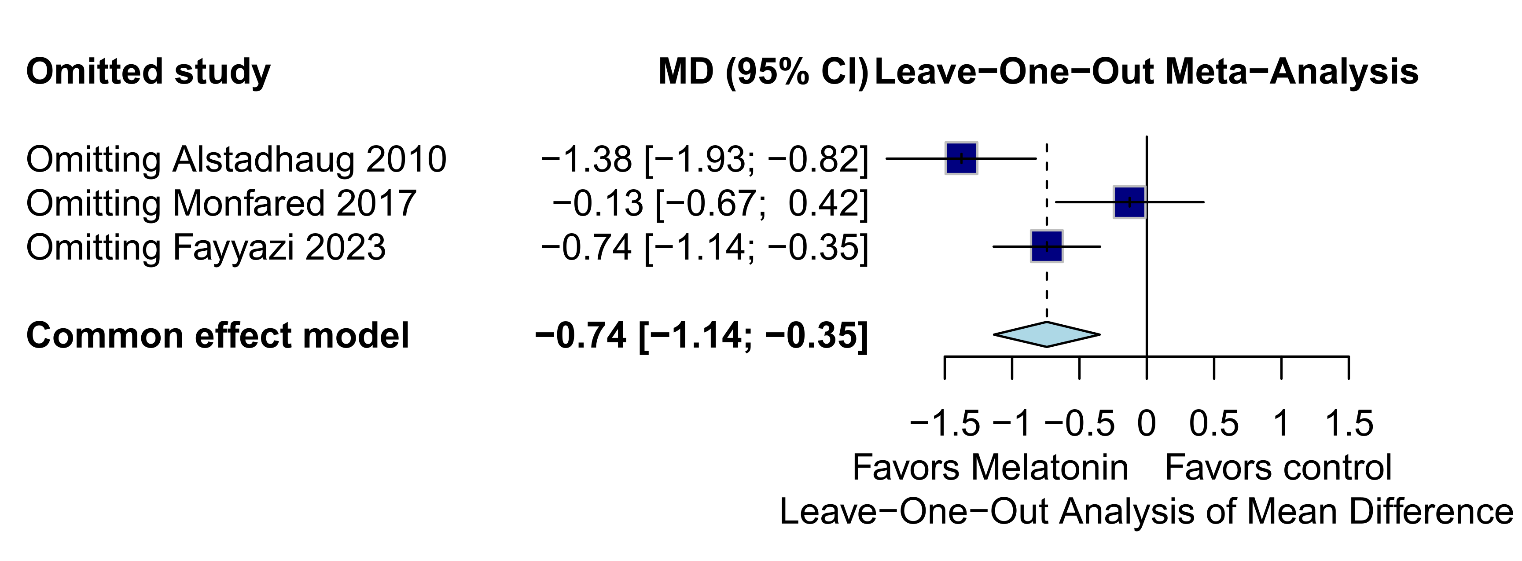
***

**Supplementary Figure 5: Sensitivity analysis migraine severity.**

**
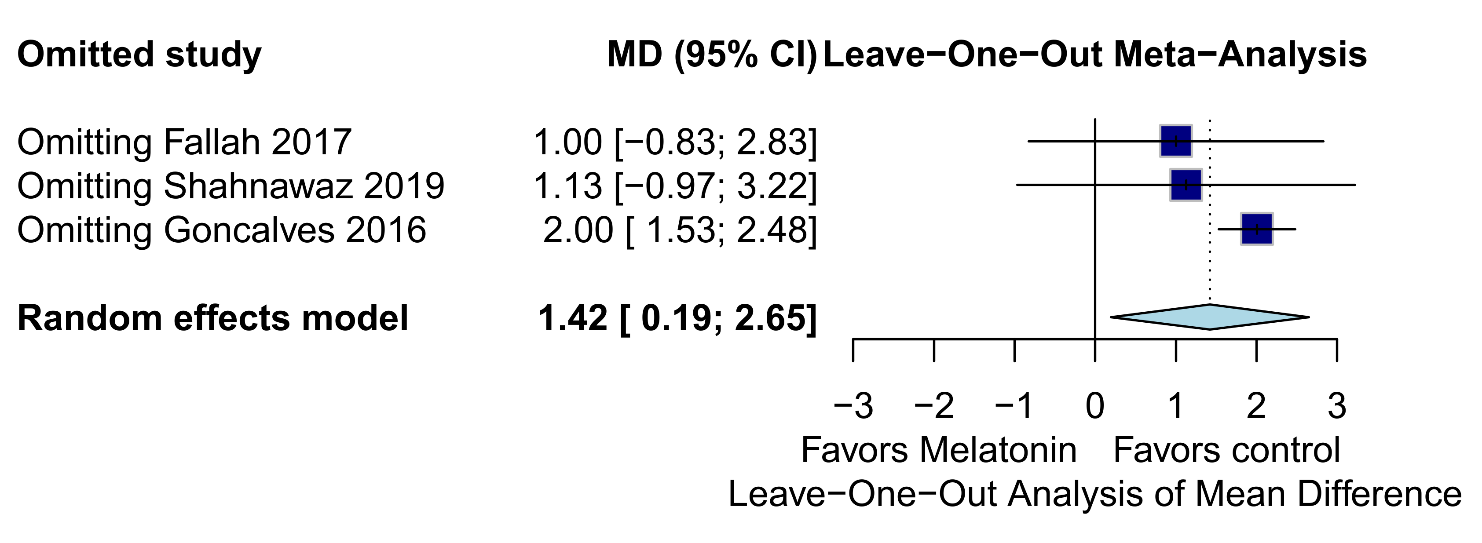
**

***Supplementary Figure 6: Subgroup analysis by headache type — migraine severity.***


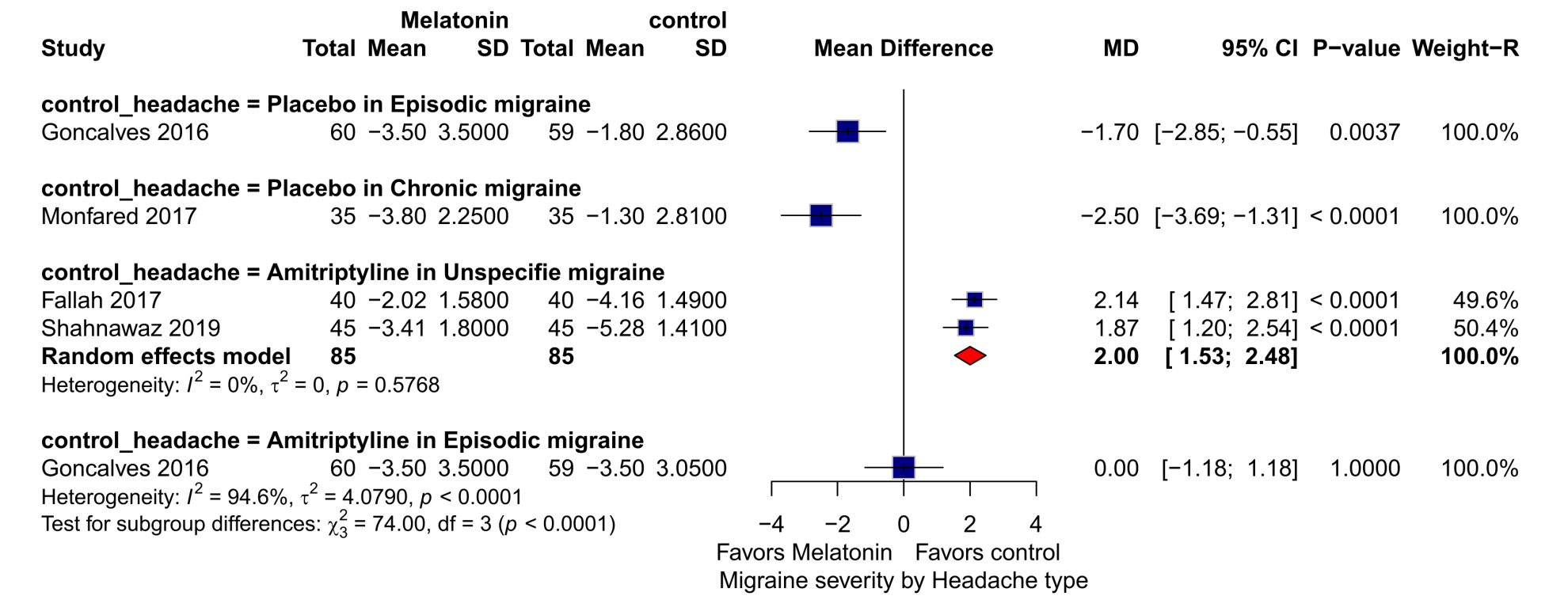


***Supplementary Figure 7: Subgroup analysis by population— migraine severity***

**
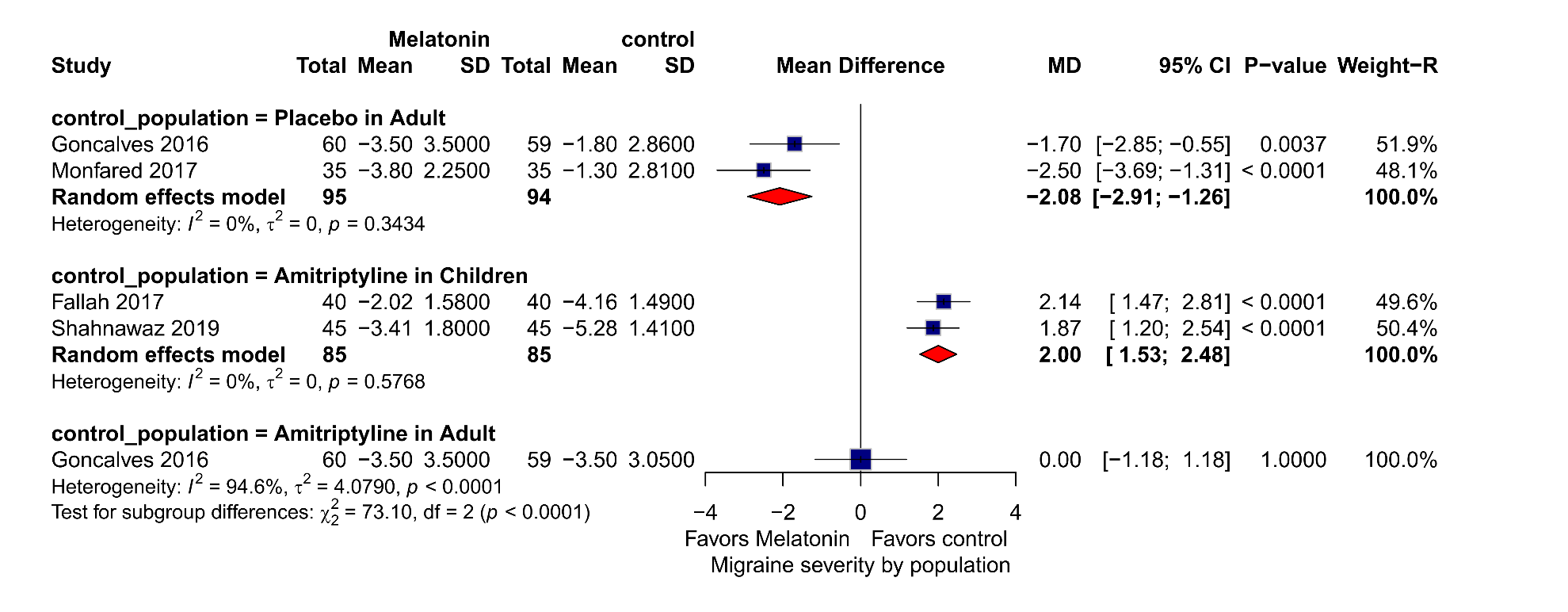
**

***Supplementary Figure 8: Subgroup analysis by population — migraine disability (MIDAS).***


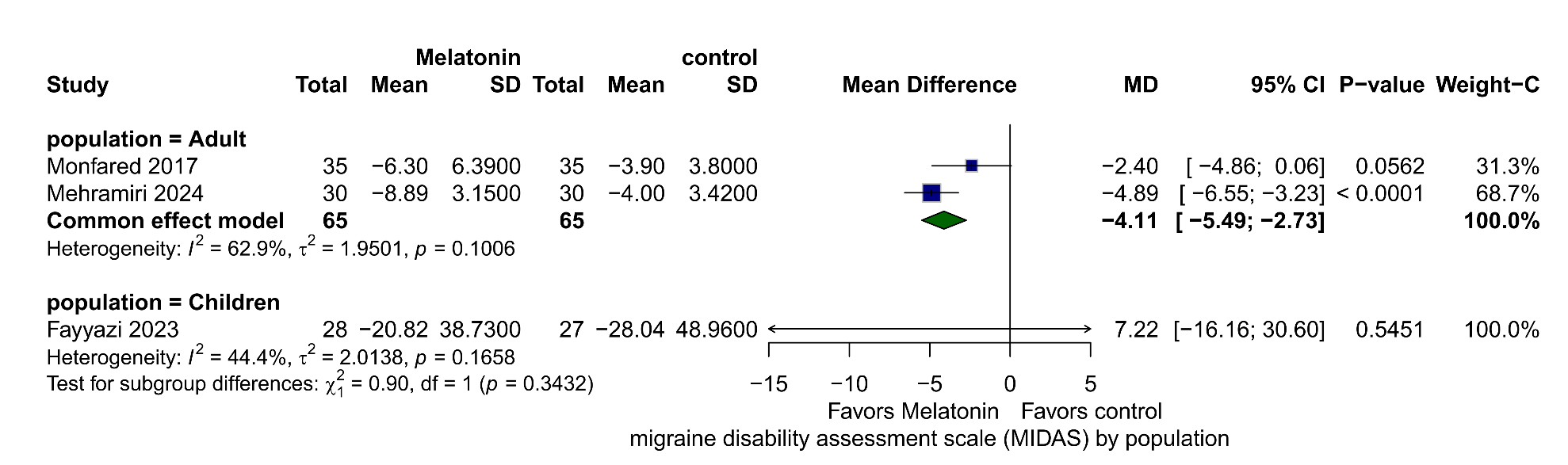


***Supplementary Figure 9: Subgroup analysis by headache type - migraine disability (MIDAS)***


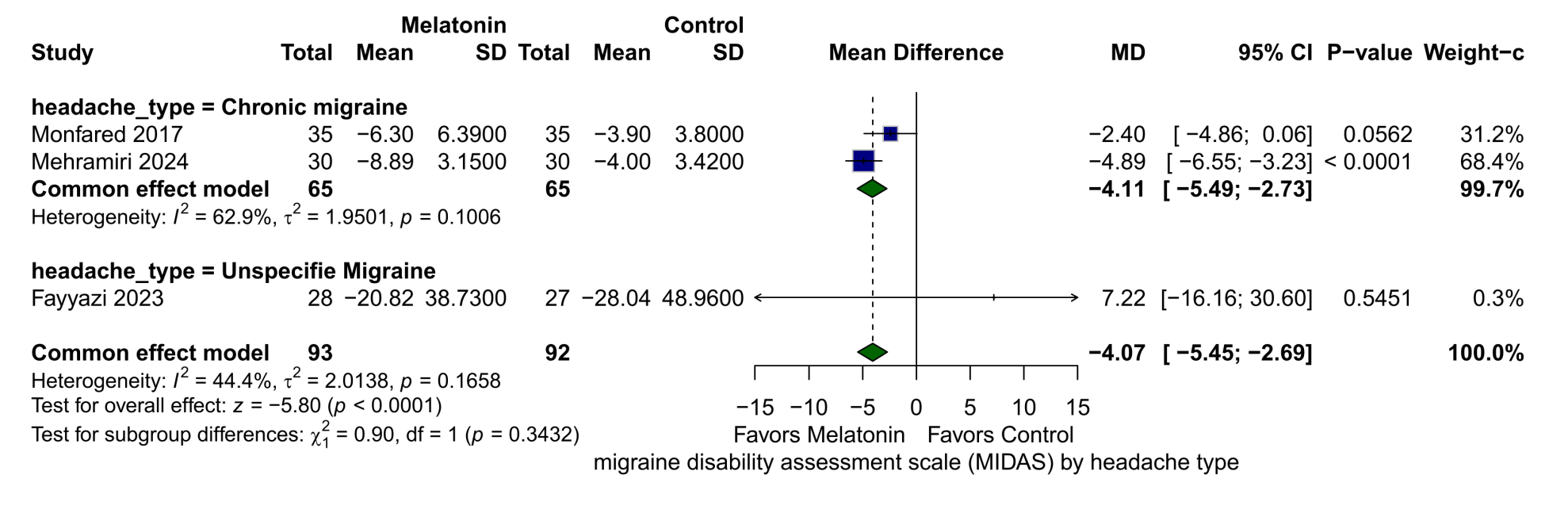


***Supplementary Figure 10: Subgroup analysis by population — response rate***


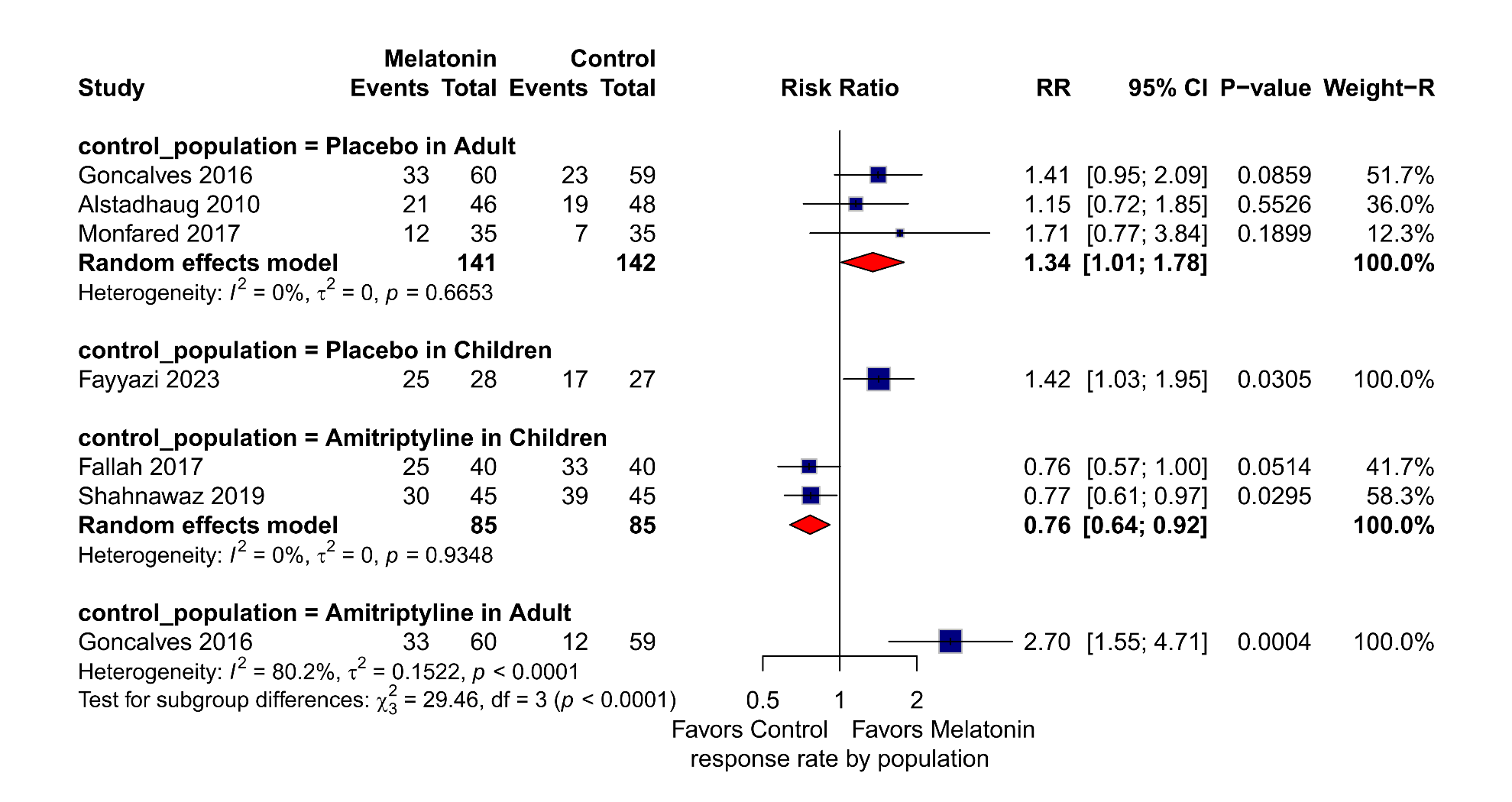


***Supplementary Figure 11: Sensitivity analysis —response rate.***


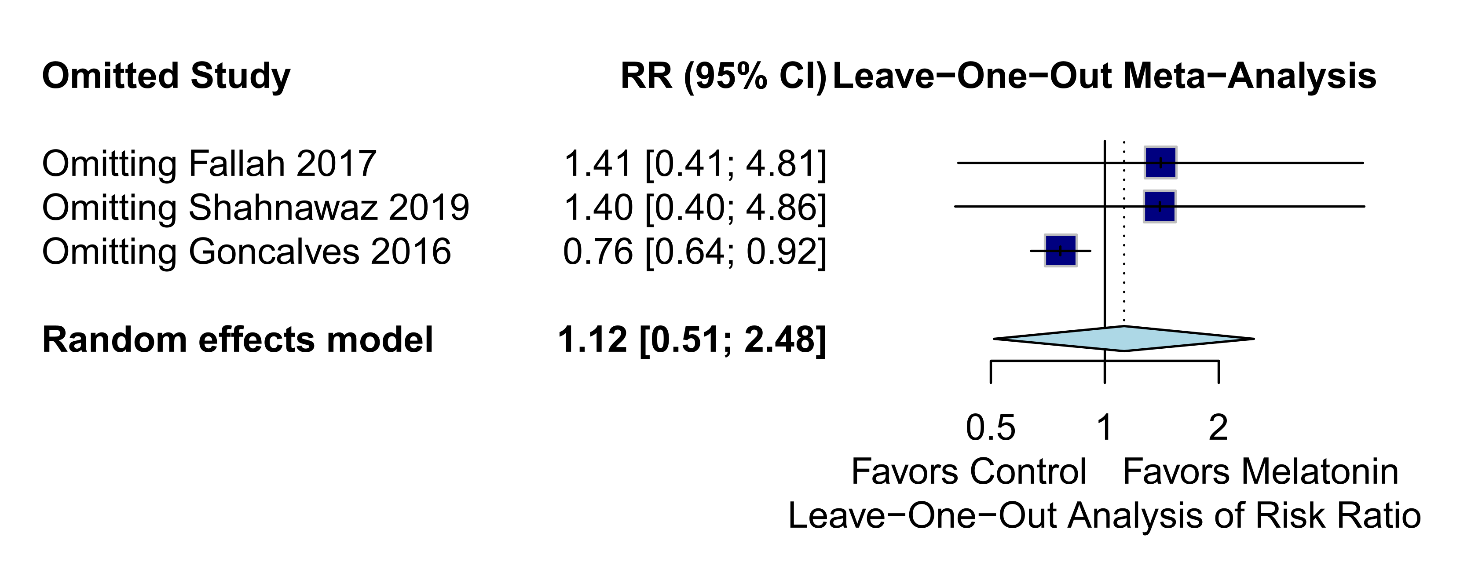


***Supplementary Figure 12: Subgroup analysis by headache type — response rate.***


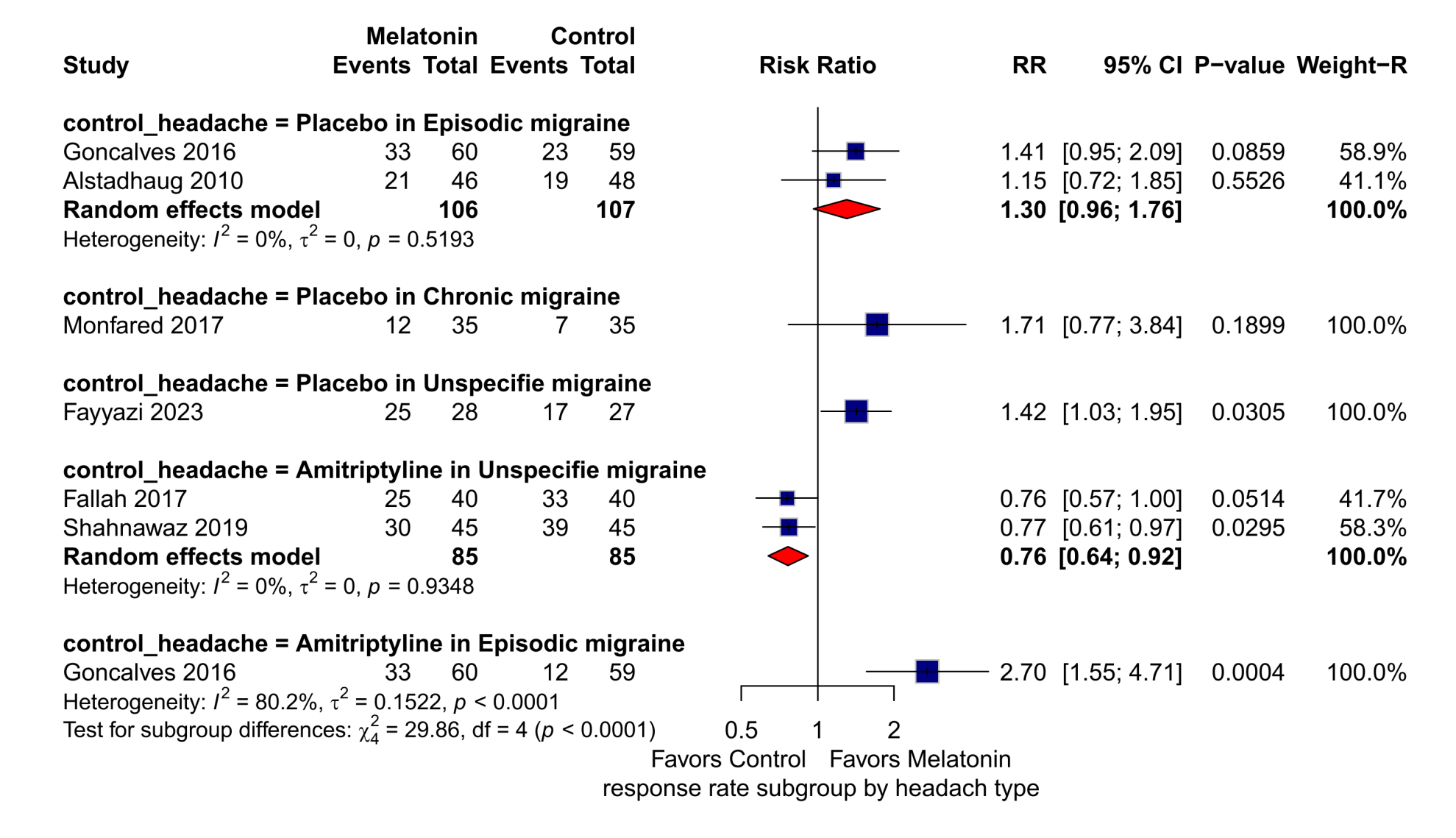

Supplement: Supplementary file 1 — Supplementary Material 1 [file 11916_2025_1461_MOESM1_ESM.docx]
